# Letter to the editor “Identification of MSC-AS1, a novel lncRNA for the diagnosis of laryngeal cancer”

**DOI:** 10.1007/s00405-020-06543-1

**Published:** 2021-01-02

**Authors:** Yuan-Fei Shi, Wan-Zhuo Xie

**Affiliations:** Department of Hematology, The First Affiliated Hospital of Medical School of Zhejiang University, No. 79 Qingchun Road, Hangzhou, 310003 Zhejiang China

Dear Editor:

With great interest, we read a recent study about a novel lncRNA (MSC-AS1) for the diagnosis of laryngeal cancer [1]. Combined a bioinformatics analysis with RT-qPCR, Liu and colleagues developed a lncRNA–miRNA–mRNA ceRNA network and identified lncRNA MSC-AS1 as a potential biomarker in laryngeal cancer. However, several aspects may better be further discussed.

**Fig. 1:**
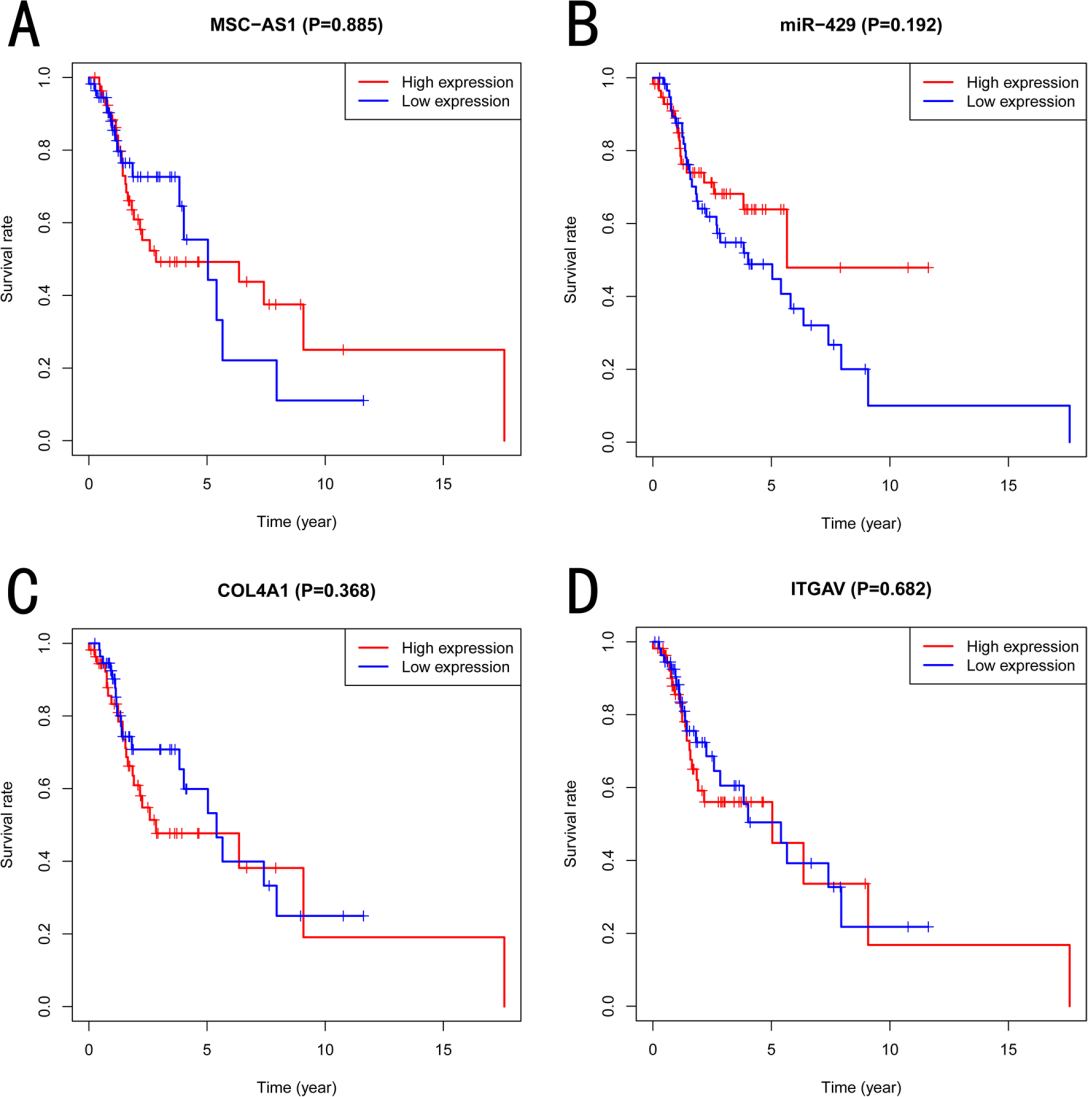
Survival analyses of the ceRNA network (MSC-AS1, miR-429, COL4A1 and ITGAV) based on the TCGA database. **a** MSC-AS1. **b** miR-429. **c** COL4A1. **d** ITGAV. The current results indicated that there was no significant difference in the survival analyses of MSC-AS1 (*P* = 0.89), miR-429 (*P* = 0.19), COL4A1 (*P* = 0.37) and ITGAV (*P* = 0.68)

As is shown in the part of the authors’ results, a ceRNA network containing MSC-AS1, miR-429, COL4A1, and ITGAV was constructed. Though their results were generated integrating the The Cancer Genome Atlas (TCGA) database and the Gene Expression Omnibus (GEO) database, they solely analyzed the expression of various genes (mRNAs, miRNAs, and lncRNA). Why not consider applying survival analyses to the validation of this study? It’s always been known that the TCGA database can provide patients’ clinical data [2, 3]. Furthermore, the TCGA database contains detailed survival data of 111 laryngeal cancer patients. Thus, we strongly suggest that the authors introduce survival analyses to validate these genes of the ceRNA network. In our Fig. 1a–d, we found that there was no significant difference in the survival analyses of MSC-AS1, miR-429, COL4A1, and ITGAV. Thus, our results indicated that Liu Y et al. novel ceRNA network still needs to further explored and the current conclusion may better be applied cautiously.

Moreover, the authors deemed that the MEblue module was positively correlated with clinical stage. But actually, we found that the MEpurple module (0.25, *P* = 0.005) should be more correlated with clinical stage in the authors’ Figure 2C, not the MEblue module (0.21, *P* = 0.02) [4, 5]. Thus, it is inappropriate to apply the data of the MEblue module to conduct the subsequent analyses.

Therefore, the current conclusion of Liu et al. still needs to be further validated. And we were looking forward to their positive responses.
